# Spectral and Structural Properties of High-Quality Reduced Graphene Oxide Produced via a Simple Approach Using Tetraethylenepentamine

**DOI:** 10.3390/nano12081240

**Published:** 2022-04-07

**Authors:** Abedalkader Alkhouzaam, Haneen Abdelrazeq, Majeda Khraisheh, Fares AlMomani, Bassim H. Hameed, Mohammad K. Hassan, Mohammad A. Al-Ghouti, Rengaraj Selvaraj

**Affiliations:** 1 Mechanical Engineering Program, Texas A&M University at Qatar, Doha P.O. Box 23874, Qatar; 200602139@student.qu.edu.qa; 2 Department of Chemical Engineering, College of Engineering, Qatar University, Doha P.O. Box 2713, Qatar; ha082881@student.qu.edu.qa (H.A.); falmomani@qu.edu.qa (F.A.); b.hammadi@qu.edu.qa (B.H.H.); 3 Center of Advanced Material (CAM), Qatar University, Doha P.O. Box 2713, Qatar; mohamed.hassan@qu.edu.qa; 4 Environmental Science Program, Department of Biological and Environmental Sciences, College of Arts and Sciences, Qatar University, Doha P.O. Box 2713, Qatar; mohammad.alghouti@qu.edu.qa; 5 Department of Chemistry, College of Science, Sultan Qaboos University, Muscat 123, Oman; rengaraj@squ.edu.om

**Keywords:** reduced graphene oxide, tetraethylenepentamine, amine-functionalized GO, characterization

## Abstract

A simple temperature-assisted solution interaction technique was used to functionalize and reduce graphene oxide (GO) using tetraethylenepentamine (TEPA) with less chemicals, low temperature, and without using other reducing agents. GO nanosheets, produced using a modified Hummers’ method, were functionalized using two different GO:TEPA ratios (1:5 and 1:10). The reduction of GO was evaluated and confirmed by different spectroscopic and microscopic techniques. The FTIR and XPS spectra revealed that most of the oxygenated groups of GO were reduced. The emergence of amide groups in the XPS survey of the rGO-TEPA samples confirmed the successful reaction of TEPA with the carboxyl groups on the edges of GO. The replacement of the oxygenated groups increased the carbon/oxygen (C/O) ratio of GO by approximately 60%, suggesting a good reduction degree. It was found that the I_2D_/I_D+D′_ ratio and the relative intensity of the D″ band clearly increased after the reduction reaction, suggesting that these bands are good estimators for the reduction degree of GO. The morphological structure of GO was also affected by the reaction with TEPA, which was confirmed by SEM and TEM images. The TEM images showed that the transparent GO sheets became denser and opaque after functionalization with TEPA, indicating an increase in the stacking level of the GO sheets. This was further confirmed by the XRD analysis, which showed a clear decrease in the d-spacing, caused by the removal of oxygenated groups during the reduction reaction.

## 1. Introduction

Since its first successful production in 2004, graphene has become a hot research topic, owing to its unique properties among other nanomaterials. Graphene nanosheets that are transparent possess high mechanical strength and resistance, and are considered to be the thinnest 2D material discovered [1,2]. Graphene also possesses interesting thermal, electronic, chemical, and optical properties that make it an ideal material for a wide range of applications, such as sensors, catalysis, adsorbents, conductive coatings, semiconductors, and optoelectronics [3,4]. Many techniques have been explored for the production of high-quality graphene nanosheets, such as chemical vapor deposition, mechanical exfoliation, and graphene oxide (GO) reduction [5]. Among them, GO reduction has attracted much interest in the past few years for the production of reduced graphene oxide (rGO) materials, which act as an ideal alternative to graphene in various applications. GO, which is typically produced by graphite oxidation, is considered to be an active site-rich modifier, due to the presence of several oxygenated functional groups in its basal plane [6]. However, the oxidation of graphite lowers the stacking level of the graphene sheets and breaks down the π-π conjugation between them, resulting in poor electrochemical properties [7]. GO can recover graphene-like properties through successful reduction that eliminates most of the oxygenated functional groups between the GO sheets and increases their stacking level. The reduction of GO can be achieved by different methods, such as chemical reduction [8,9], photoreduction [10,11], hydrothermal reduction [12,13], and electrochemical reduction [14]. 

The chemical reduction reaction is commonly used, due to the availability of several reductants, such as hydrazine, hydrohalic acids, dimethylhydrazine, sodium borohydride, and amines [1,3]. In this context, the amination of GO with various amines remains one of the most effective ways to reduce GO and to enhance its properties. Amines with various functional groups can be used as reductants and functionalization agents to stabilize GO nanosheets, and to enhance their stability and electrochemical properties [5]. The successful selection of an amine can reduce GO and, at the same time, decorate it with specific functional groups to tackle the desired application. Therefore, various amines, such as dodecylamine [15], polydopamine [16], butylamine [17], and melamine [18], have been used as GO reductants for different applications. These applications include membrane enhancement [19], sensing applications [20], anti-corrosion coatings [21], and adsorption [22]. 

In this work, tetraethylenepentamine (TEPA), a low-cost and low-toxicity compound, was used to simultaneously reduce and functionalize GO nanosheets. GO reduction with TEPA has been conducted and reported in some studies. Jayabal et al. [7] used TEPA and hydrazine hydrate via a hydrothermal process at 180 °C. Hydrothermal reduction was also preformed by Han et al. [23], excluding the use of hydrazine hydrate. Liu et al. [24] introduced an ultrasound-assisted technique to reduce and functionalize GO with TEPA, in the presence of N-(3-dimethylaminopropyl)-N′-ethylcarbodiimide hydrochloride (EDC) and hydroxybenzotriazole (HOBT) as coupling agents. Ribeiro et al. [25] conducted GO-TEPA functionalization using a microwave radiation-assisted reaction at 120 °C. As clearly observed, the reported studies used high temperatures and/or reducing agents to facilitate the reduction process. Herein, we report TEPA-induced GO reduction using a simple temperature-assisted solution interaction technique, using less reactants, lower temperatures, and without using other catalytical or reducing agents. Two different GO:TEPA ratios were used to produce the rGO-TEPA nanosheets with different reduction degrees. The functionalization reaction was simply conducted using GO–water dispersion and a TEPA–ethanol solution, under reflux conditions. Various spectral, structural, and morphological analytical techniques were used to study the efficiency of the reduction reaction, and to explore the properties of these nanosheets. 

## 2. Experimental Methods

### 2.1. Materials

Graphite flakes with a −10 mesh flake size (99.9%) were supplied by Alfa Aesar (Waltham, MA, USA). H_2_SO_4_ (95%) and KMnO_4_ (99%) were obtained from Fisher Scientific (Waltham, MA, USA). H_3_PO_4_ (99%), H_2_O_2_ (30%), ethanol (99.7–100%) and HCl (35–38%) were supplied by BDH middle east, Dubai, UAE. Tetraethylenepentamine (TEPA) was purchased from Sigma Aldrich (St. Louis, MO, USA). The ELGA PURELAB water purifier (Lane End, UK) was used to produce the deionized water (DI). All materials were utilized as purchased, without further purification.

### 2.2. GO Nanosheet Preparation

GO nanosheets were synthesized using the conventional Hummers’ method, with a few modifications, as reported earlier [26]. Briefly, 24 mL of sulfuric acid (H_2_SO_4_) and 6 mL of phosphoric acid (H_3_PO_4_) (volume ratio 4:1) were mixed in an ice bath for a few minutes. Then, graphite flakes (1 g) and KMnO_4_ (3 g) were gradually loaded into the acid mixture under stirring conditions. The reaction was then shifted to a heated oil bath (95 ± 2 °C) and stirred for 30 min. The mixture was then diluted with 50 mL of deionized water (DI) and kept under the same conditions for 150 min. The reaction was then terminated by the addition of 150 mL of DI and 10 mL of H_2_O_2_. The mixture was then diluted with 20% HCl solution and centrifuged at 5000 rpm for 15 min, and the supernatant was removed. The residuals were then washed and centrifuged several times with DI, until the pH was neutralized. Finally, the prepared samples were dried in the oven at 80 °C for two days. 

### 2.3. GO Reduction and Functionalization

**Figure 1:**
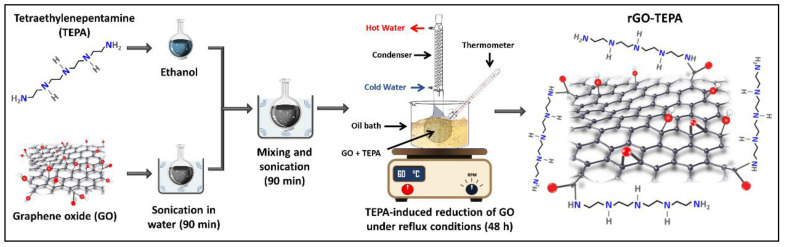
Schematic representation of the TEPA-induced GO reduction and the predicted chemical structure of rGO-TEPA.

GO nanosheets were simultaneously functionalized and reduced using the temperature-assisted reflux technique. In a typical experiment, 200 mg of GO nanosheets are ultrasonicated in DI for 90 min, to ensure that the GO nanosheets are well dispersed. Certain amounts of TEPA (0.5 mL for rGO-TEPA1 and 2 mL for rGO-TEPA2) were dissolved in 50 mL of ethanol. The GO suspension and the TEPA solution were then mixed in a round-bottom flask and ultrasonicated for 90 min. The mixture was then refluxed in an oil bath at 60 °C for 48 h. The rGO-TEPA powders were obtained by centrifugation at 5000 rpm for 15 min, and were then washed/centrifuged three times with ethanol to remove the unreacted species. The products were then dried in the oven at 70 °C overnight. A schematic representation of the TEPA-induced reduction of GO and the expected rGO-TEPA structure is shown in Figure 1. 

### 2.4. Characterization Techniques

Different characterization techniques were employed to analyze the oxidation, reduction, and functionalization degree of the GO nanosheets. The morphological structures of the prepared GO and rGO samples were analyzed using scanning electron microscopy (SEM, JEOL model JSM-6390LV, Tokyo, Japan) and transmission electron microscopy (TEM, FEI TF20, 200 kV, Oregon, OR, USA). Prior to the TEM analysis, the GO and rGO-TEPA samples were dispersed in isopropanol in an ultrasonic bath for 20 min, and 2 drops of the dispersion were drop-casted using a micropipette (10 µL) onto a carbon film on copper (300 mesh). The FTIR spectra, in the range of 400–4000 cm^−1^, were recorded with a Spectrum 400 FTIR spectrometer from PerkinElmer (Waltham, MA, USA) using a UATR. The Raman spectra were recorded using a Thermo Fisher Scientific DXR Raman Microscope (Thermo Scientific™, Waltham, MA, USA) with a wavelength of 532 nm, 40 times scan, and a laser power of 10, using a 10X microscope objective. The compositional properties were analyzed using a CHNO elemental analyzer (Flash 2000, Thermo Scientific™, Waltham, MA, USA) and X-ray photoelectron spectroscopy (XPS, Kratos AXIS Ultra DLD, Manchester, UK, with Al-Kα source, X-ray power of 15 Kv, 20 mA and over the 0–1200 eV range). X-ray diffraction analysis (XRD) was conducted using an Empyrean PANalytical diffractometer (Malvern Panalytical B.V., Eindhoven, Netherlands). The thermal stability of the GO and rGO-TEPA samples was evaluated using a thermogravimetric analyzer (TGA) from PerkinElmer (Waltham, MA, USA) under nitrogen gas and with a 10 °C/min heating rate.

## 3. Results and Discussion

### 3.1. FTIR and Raman Spectral Properties

**Figure 2:**
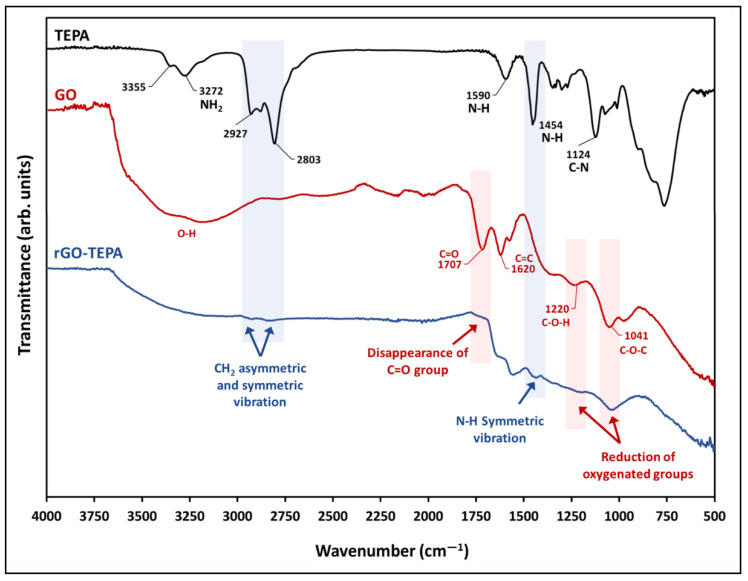
FTIR-UATR spectra of TEPA, pristine GO, and rGO-TEPA nanohybrid.

Figure 2 shows the FTIR spectra of TEPA, the pristine GO nanosheets, and the prepared rGO-TEPA nanohybrids. The spectra of the pristine GO confirm the graphite oxidation and the formation of GO, due to the emergence of several oxygen-containing functional groups, including the stretching vibration of the carbonyl groups located on the edges of the GO nanosheets at ~1707 cm^−1^, the hydroxyl group bending vibrations at ~1220 cm^−1^, and the epoxy stretching vibration at ~1041 cm^−1^. The hydroxyl group around ~3400 cm^−1^ is attributed to the residual water molecules between the GO nanosheets, while the C=C vibration around 1620 cm^−1^ is related to the unoxidized graphene [26,27]. The black line in Figure 2 represents the TEPA spectra, and shows the characteristic functional groups, including the amino groups around 3355 cm^−1^ and 3272 cm^−1^, and the CH_2_ symmetric and asymmetric stretching vibration bands around 2803 cm^−1^ and 2927 cm^−1^, respectively [28,29]. The band around 1124 cm^−1^ corresponds to the C–N stretching vibration, while the bands around 1454 cm^−1^ and 1590 cm^−1^ correspond to the N–H bending vibration [28,30]. The spectra of rGO-TEPA (the blue line) confirm the functionalization of GO, through the presence of new bands around 1454 cm^−1^, 2803 cm^−1^, and 2927 cm^−1^, corresponding to the N–H symmetric vibration, and the CH_2_ symmetric and asymmetric stretching vibrations, respectively. Moreover, the spectra show a clear reduction of the oxygenated groups around 1220 cm^−1^ and ~1041 cm^−1^, accompanied by the disappearance of the carbonyl group around 1707 cm^−1^, which confirms the reduction of GO to rGO. These findings are in good agreement with the anticipated rGO-TEPA structure presented in Figure 1. 

**Figure 3:**
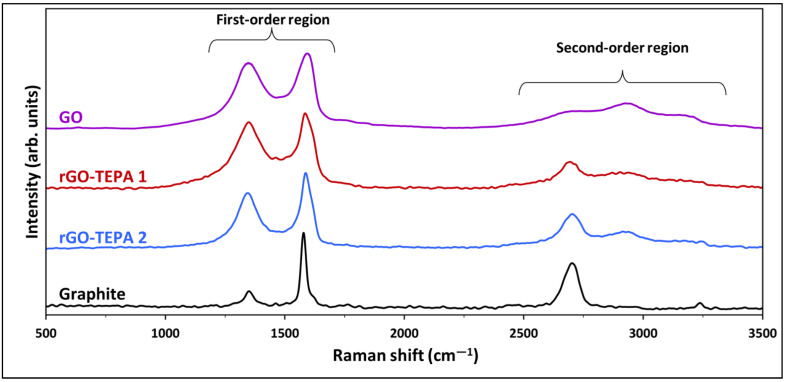
Raman spectra of the graphite, pristine GO, and rGO-TEPA1 and rGO-TEPA2 nanohybrids. The spectra were normalized by their highest intensity for clarity.

The Raman spectra method is one of the most powerful techniques used to analyze the properties of graphene-based materials, and to detect the defects and the changes in their layer structure [31]. The Raman spectra of graphite, GO and the rGO samples are presented in Figure 3, and exhibit two distinct regions, the first-order and second-order regions, which are identical for graphene-based materials. The spectra of the raw graphite exhibit two distinct peaks around 1580 and 2700 cm^−1^, corresponding to the G and 2D (G′) bands, respectively, which are considered to be the prominent features of a graphene monolayer [32,33]. However, after the oxidation reaction, an intense peak appears at ~1350 cm^−1^ in the first-order region, in addition to two other peaks in the second-order region, confirming the formation of GO. After GO functionalization with TEPA, these peaks were reduced, which confirms the reduction of GO. 

**Figure 4:**
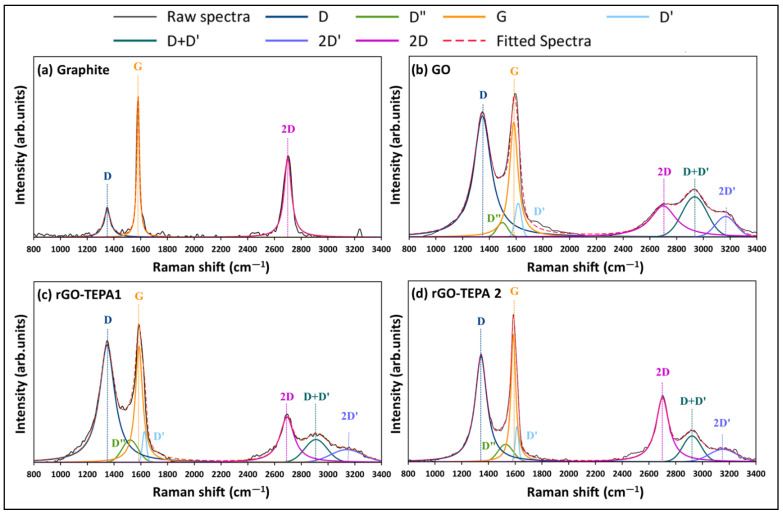
The deconvolution and peak fitting of the Raman spectra of (**a**) graphite, (**b**) pristine GO, and (**c**) rGO-TEPA1 and (**d**) rGO-TEPA2 nanohybrids.

Following our previous work [26], the Raman spectra of all the samples were deconvoluted and fitted to enable a better understanding of the spectra and the properties of the associated samples. The spectra deconvolution and peak fitting are presented in Figure 4. Two intense peaks were observed in the spectra of graphite (G and 2D), with a small peak in the first-order region, corresponding to the D band at ~1350 cm^−1^. The ratio of the integrated intensities of the D and G bands (I_D_/I_G_) and of the pristine graphite was ~0.5. After the oxidation of graphite (Figure 4b), the intensity of the D band was significantly enlarged, with an I_D_/I_G_ ratio of 2.19. Moreover, a broad shoulder formed between the D and G bands, which is attributed to the presence of D″ and D′ peaks at ~1497 and 1615 cm^−1^, respectively. These peaks were reported with different GO-based materials [15,27]. The deconvolution of the second-order region of the GO spectra showed a decrease in the relative intensity of the 2D band, accompanied by the emergence of two additional peaks around 2934 and 3166 cm^−1^, corresponding to the D+D′ and 2D′ bands, respectively. Similarly, the relative intensities of the first- and second-order bands were affected by the reduction of GO with TEPA, as demonstrated in Figure 4c,d. A slight reduction in the I_D_/I_G_ ratios of rGO-TEPA1 and rGO-TEPA2 was observed (2.16 and 2.09, respectively). However, the relative intensity of the D″ band increased with the reduction of GO. The percentage of D″ band in the first-order region was approximately 3.1%, which increased to 8.1% and 8.2% with rGO-TEPA1 and rGO-TEPA2, respectively. The deconvolution of the second-order region showed that the intensity of the 2D band increased with the reduction degree of GO, while the D+D′ band behaved oppositely. The I_2D_/I_D+D′_ ratio, calculated from the peak fitting, showed a clear increase from 1.21, for the pristine GO, to 1.90 and 2.68 for rGO-TEPA1 and rGO-TEPA2, respectively. These findings confirm the reduction of GO, and imply that the relative intensities of the D″, 2D, and D+D′ bands are good estimators for the reduction degree of GO. Tables S1 and S2 in the Supplementary Information collect the band parameters obtained from the first- and second-order spectra fittings, respectively.

### 3.2. Structural and Morphological Properties

**Figure 5:**
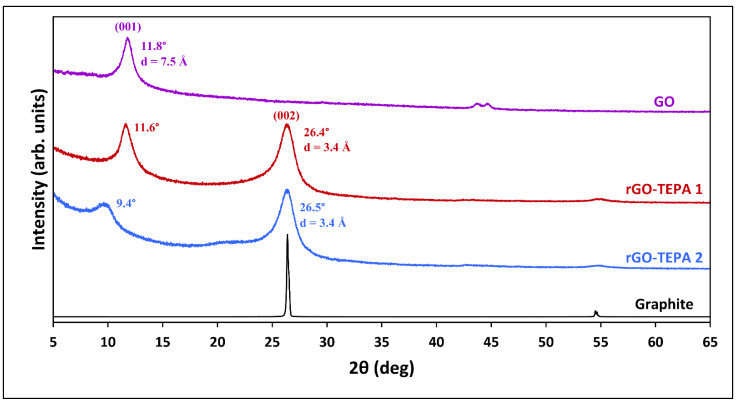
XRD patterns of the graphite, GO, and rGO-TEPA1 and rGO-TEPA2 nanohybrids. The spectra were normalized by their highest intensity for clarity.

The structural change before and after GO reduction was studied using the XRD analysis. Figure 5 shows the XRD patterns of the pristine GO and the functionalized samples. An intense diffraction peak was observed in the pristine GO pattern at 11.8°, corresponding to the 001 plane of the GO crystal structure. The d-spacing of the 001 plane (d_001_), estimated using the Bragg equation, is 7.5 Å, which is close to the values reported in the literature [6,34]. Previous studies have reported that the d_001_ spacing of GO is affected by the water molecules trapped between the GO layers, and ranges between 6.1 Å and 12 Å for dry and hydrated GO, respectively [8,34]. The XRD patterns of the reduced samples showed that the 001 peak was reduced and shifted to a lower diffraction angle. Similar observations were previously reported with different types of amine-functionalized GO [6,35]. Another peak emerged in the patterns of both rGO samples at around 26.5°, corresponding to the 002 plane, confirming the reduction of GO. Moreover, the calculated interlayer spacing (d_002_) for both rGO-TEPA1 and rGO-TEPA2 was found to be 3.4 Å, which is identical to the thickness of a single graphene layer [36,37]. These findings suggest a good reduction degree of GO and the high quality of the rGO nanosheets. 

**Figure 6:**
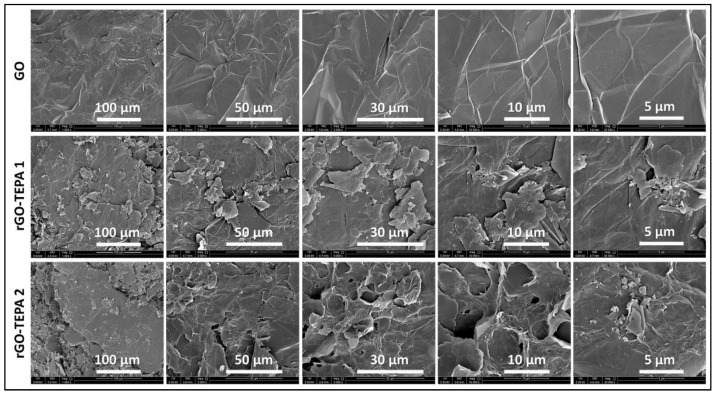
SEM images of the pristine GO, rGO-TEPA1 and rGO-TEPA2.

**Figure 7:**
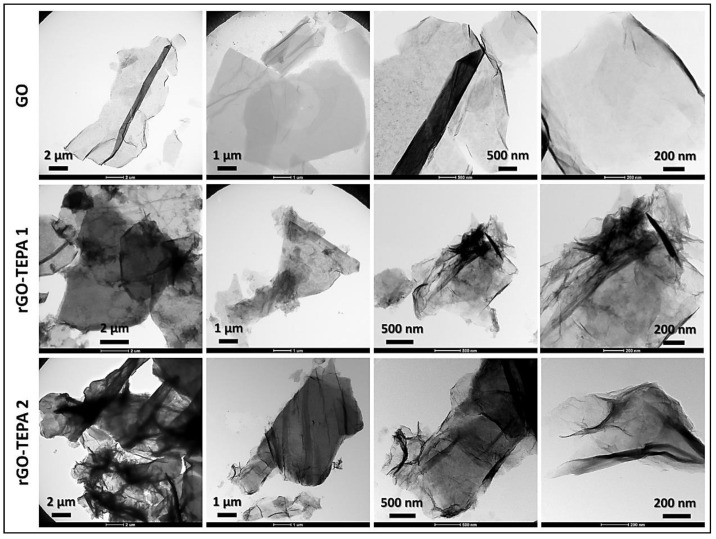
TEM images of the pristine GO, rGO-TEPA1 and rGO-TEPA2.

**Figure 8:**
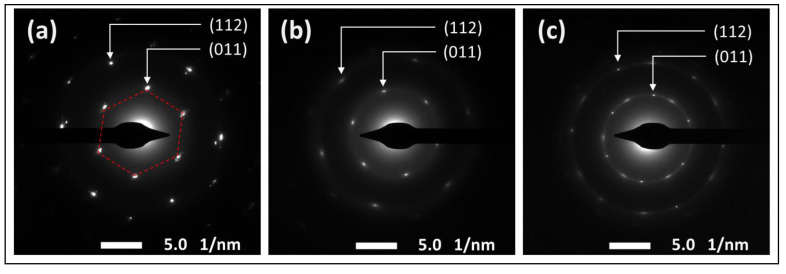
SAED diffraction patterns of (**a**) the pristine GO, (**b**) rGO-TEPA1 and (**c**) rGO-TEPA2.

The effect of the functionalization reaction on the morphological structure of GO was investigated using SEM and TEM techniques. The SEM images in Figure 6 show the sharp, clear, wrinkled, and regular structure of the pristine GO. This morphological structure is typical for GO nanosheets, and is caused by the oxidation of a graphite cluster [24]. However, the images of the reduced samples show randomly aggregated sheets with a rougher surface and an irregular structure. This structure can be attributed to the intercalation of TEPA molecules between the GO flakes, confirming the amine functionalization and reduction of GO with TEPA. A similar structure has been reported with several amine-rGOs [15,38,39]. The reduction reaction can be further confirmed by the TEM images presented in Figure 7. The images of the pristine GO show highly transparent sheets with sharp edges. The high transparency is attributed to the low stacking level of the GO sheets, due to their high oxygen content [26]. With the low loading of TEPA (1 mL), the sheets exhibited dense and opaque surfaces, and became denser and darker when the TEPA concentration was doubled. This can be explained by the increase in the stacking level of the graphene sheets, due to the reduction in the interlayer spacing, as confirmed by the XRD results. The selected area electron diffraction (SAED) patterns presented in Figure 8 confirm these findings and the structural change due to GO reduction. The SAED patterns of the pristine GO show clear and sharp hexagonal spots, confirming the typical structure of a monolayer graphene (Figure 8a). In contrast, the diffused diffraction patterns shown in Figure 8b,c suggest that the graphene sheets are randomly stacked with a lower stacking level, due to the removal of the oxygenated groups, confirming the formation of rGO [40,41].

### 3.3. XPS and Compositional Properties

**Figure 9:**
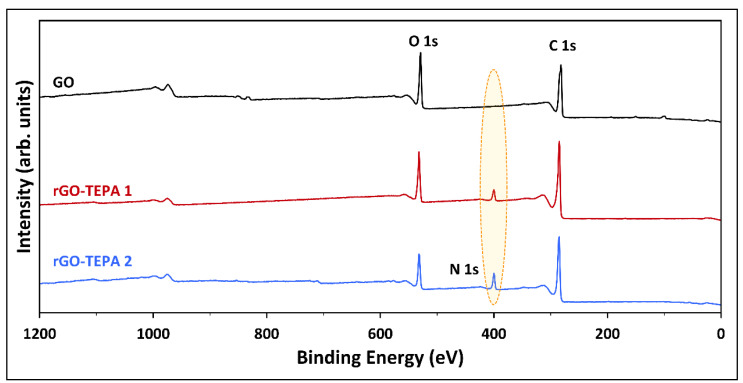
XPS survey of the pristine GO, and rGO-TEPA1 and rGO-TEPA2 nanohybrids.

The pristine GO and the rGO samples were characterized by XPS to investigate the effect of TEPA functionalization on the surface compositions of GO. The extended XPS surveys and the surface compositions of the prepared samples are presented in Figure 9 and Table 1, respectively. The XPS survey of the pristine GO revealed the presence of intense peaks at 282 and 529 eV, corresponding to C 1s and O 1s, respectively. The surface atomic compositions of the pristine GO were 77.79 at.% and 22.06 at.% for C 1s and O 1s, respectively, with a carbon/oxygen (C/O) ratio of 3.53. The successful amination was confirmed by the emergence of the N 1s peak, with compositions of 4.36 at.% and 7.73 at.% in the XPS spectra of rGO-TEPA1 and rGO-TEPA2, respectively. Moreover, the atomic C/O ratio increased to 4.43 and 5.59 with rGO-TEPA1 and rGO-TEPA2, respectively, suggesting a good reduction degree. These findings were further confirmed by the CHNO elemental analysis. The weight compositions of the pristine and reduced GO samples are listed in Table 2. The increase in nitrogen and hydrogen content confirms the attachment of the amine groups on GO, which is consistent with the anticipated rGO-TEPA structure illustrated in Figure 1. It is worth mentioning that XPS provides information about the surface compositions of the surveyed area, while the CHNO results represent the bulk compositions [26]. This explains the difference between the compositions obtained by each characterization technique, which has been reported in some studies [26,34].

**Figure 10:**
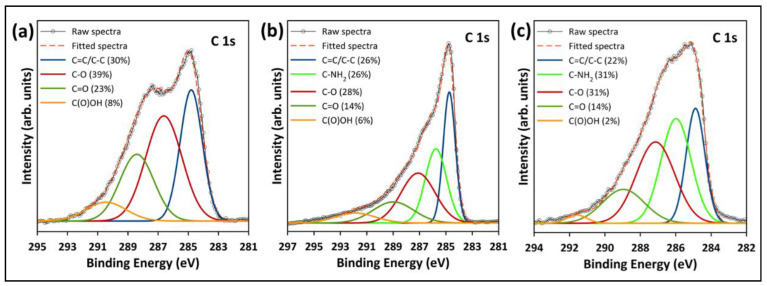
High-resolution XPS deconvolution of C 1s for (**a**) the pristine GO, (**b**) rGO-TEPA1, and (**c**) rGO-TEPA2.

**Figure 11:**
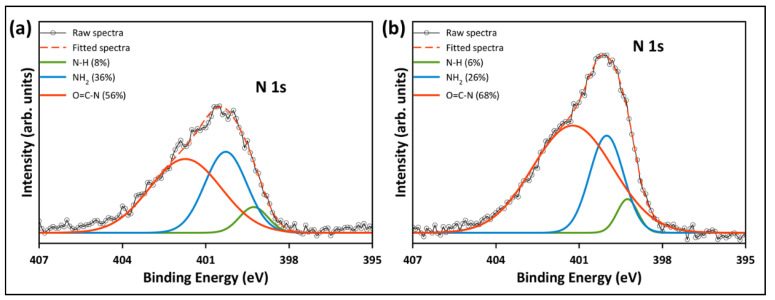
High-resolution XPS deconvolution of N 1s peak for (**a**) rGO-TEPA1 and (**b**) rGO-TEPA2.

The high-resolution XPS spectra were studied to evaluate the chemical state of the functional groups presented in the pristine and functionalized GO. Figure 10a depicts the deconvolution and fitting of the C 1s core level of the pristine GO, which resulted in four peaks around the binding energies of 284.8, 286.6, 288.4, and 290.5 eV, corresponding to the C–C/C=C, C–O, C=O, and COOH groups, respectively [26,34]. In the deconvolution of the C 1s of the functionalized samples (Figure 10b,c), another peak emerged at ~286 eV, corresponding to the C–NH_2_ group from the TEPA amine groups [7,42]. Additionally, Figure 10b,c reveals a reduction in the epoxy, carbonyl and carboxyl peaks, indicating that most of the oxygenated groups were removed during the functionalization with TEPA. The peak fitting of the N 1s core level of rGO-TEPA1 and rGO-TEPA2 is depicted in Figure 11a,b. The N 1s was deconvoluted and fitted into three peaks at ~399, 400, and 401 eV, corresponding to the secondary amine, primary amine, and amide groups, respectively [25]. The presence of the amide group provides further evidence of the successful reaction between TEPA and the carboxyl groups on the GO nanosheets. These findings are in good agreement with the FTIR spectra and the rGO-TEPA structure illustrated in Figure 1. Moreover, increasing the TEPA loading during the reaction increased the percentage of amide groups from 56% with rGO-TEPA1 to 68% with rGO-TEPA2. This caused more removal of the hydroxyl groups, which explains the higher reduction level of rGO-TEPA2 than that of rGO-TEPA1. Table S3 in the Supplementary Information collects the parameters and compositions of the functional groups obtained from the XPS deconvolution and fitting.

Table 3 compares the methods, reactants, C/O ratio, and reduction degree of the rGO-TEPA prepared in the present study with other studies in the literature. It can be clearly observed that the reported studies used either extra reducing/catalytic agents or higher temperatures to facilitate the reduction reaction. Most of these agents are hazardous and toxic. However, a good reduction degree was achieved in this work using less chemicals and lower temperatures. 

### 3.4. Thermal Stability

**Figure 12:**
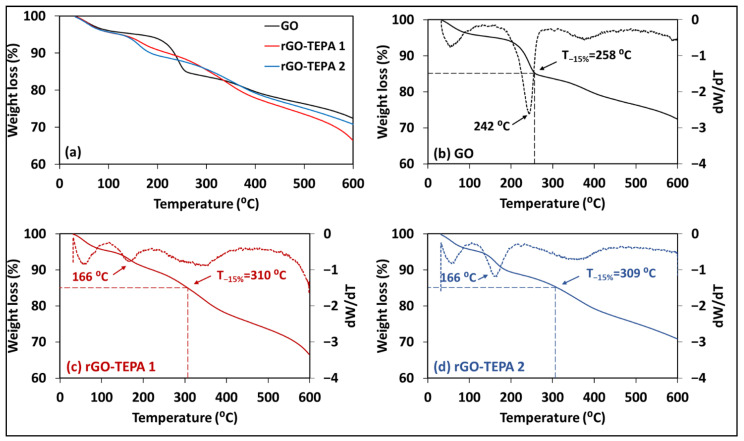
(**a**) TGA curves of the prepared samples: TGA (solid lines) and D-TGA (dotted lines) curves of (**b**) the pristine GO, (**c**) rGO-TEPA1, and (**d**) rGO-TEPA2.

The change in elemental compositions of the GO samples affects their thermal stabilities, due to the dependency of thermal decomposition on the bond dissociation energies [43]. Therefore, the thermal stability of the pristine GO, rGO-TEPA1 and rGO-TEPA2 was studied, in terms of their TGA curves, which are presented in Figure 12a. The TGA curves of the samples revealed a slight difference in their thermal stabilities. The pristine GO exhibited two weight loss regions around 100 °C and 242 °C (Figure 12b). The initial weight loss (~100 °C) is attributed to the removal of water molecules between the GO sheets, while the loss at higher temperatures is attributed to the decompositions of the unstable oxygen-containing groups [44]. The reduced GO samples followed the same behavior in the first stage, while they showed slight enhancement in their thermal stability at high temperatures (Figure 12c,d). For example, the pristine GO lost around 15% at 258 °C, as demonstrated by Figure 12b. However, the 15% loss occurred at around 310 °C for the reduced samples, as shown in Figure 12b,c. This can be explained by the removal of oxygenated groups during the reduction with TEPA, which provides further evidence for the formation of rGO. 

## 4. Conclusions

The present study investigates the effectiveness of using TEPA as a GO reducing agent, without using other catalytical or reducing agents. The GO nanosheets were successfully functionalized and reduced with TEPA, via a simple temperature-assisted solution interaction approach with low quantities of TEPA. The properties of the prepared samples were evaluated using different analytical techniques, to investigate the effectiveness of the reduction reaction. The FTIR spectral analysis revealed that GO was successfully reduced through the reduction of the carbonyl and other oxygen-containing groups. The results were further evidenced by the XPS analysis, which showed the emergence of an amide group in the spectra of the rGO-TEPA samples, indicating that TEPA reacted effectively with the carboxyl groups on the edges of GO. The Raman spectra showed the characteristic D and G bands with a high I_D_/I_G_ ratio for the pristine GO, which was slightly reduced after the reduction with TEPA. Furthermore, the Raman spectral analysis revealed that the relative intensity of the D″ band and the I_2D_/I_D+D′_ ratio of the reduced samples was much higher than those of the pristine GO. This suggests that the D″, 2D, and D+D′ bands are also good estimators for the reduction degree of GO. The structural change in the GO nanosheets was analyzed using the XRD patterns. The XRD analysis showed an emergence of the 002 plane of graphene, with a decrease in the interlayer spacing, confirming the removal of oxygenated groups between the GO sheets. This was further confirmed by the TEM images, which showed opaque rGO sheets, indicating a higher stacking level than that of the pristine GO sheets. Furthermore, the removal of oxygenated groups between the GO sheets affected their thermal stability, providing better stability of the reduced samples at high temperatures than the pristine GO. 

## Figures and Tables

**Table 1:** Surface elemental compositions of the pristine GO and rGO samples obtained by the XPS analysis.

Sample	Peak	Binding Energy (eV)	at.%	C/O (at)
GO	C 1s	284	77.79	3.53
	O 1s	530	22.06	
	S 2p	168	0.15	
rGO-TEPA1	C 1s	285	77.88	4.43
	O 1s	532	17.60	
	N 1s	400	4.36	
	S 2p	169	0.16	
rGO-TEPA2	C 1s	285	78.21	5.59
	O 1s	532	13.98	
	N 1s	400	7.73	
	S 2p	169	0.08	

**Table 2:** Weight compositions of the prepared samples obtained by the CHNO analysis.

Sample	N (wt.%)	C (wt.%)	H (wt.%)	O (wt.%)	C/O (wt%)
GO	0	57.02	1.99	40.99	1.4
rGO-TEPA1	4.60	63.31	2.82	29.27	2.2
rGO-TEPA2	6.20	66.20	3.14	24.46	2.7

**Table 3:** Comparison of the experimental conditions, chemicals used, C/O ratio and reduction degree of the rGO-TEPA prepared in this study with other studies in the literature.

GO:TEPA	Catalytic/Reducing Agent	Method and Conditions	C/O	Reduction % *	Ref.
1:100	Hydrazine hydrate	Hydrothermal reduction (180 °C, 5 h)	1.6 ^a^	-	[7]
1:5	-	Hydrothermal reduction (180 °C, 20 h)	-	-	[23]
1:10	EDC and HOBT	Ultrasound-assisted and chemical activation (40 °C, 24 h)	3.59 ^b^	7.6 ^b^	[24]
1:1500	-	Microwave radiation-assisted reaction (120 °C, 30 min)	-	-	[25]
1:10	-	Temperature-assisted solution interaction (60 °C, 48 h)	5.59 ^a^ 2.7 ^b^	36.6 ^a^ 40.3 ^b^	This work

* Reduction % with respect to the O% reduction after functionalization. ^a^ Based on the atomic compositions obtained by XPS. ^b^ Based on weight composition.

## Data Availability

Not applicable.

## Supplementary Materials

The following supporting information can be downloaded at: https://www.mdpi.com/article/10.3390/nano12081240/s1, Table S1: Bands parameters estimated from the Raman first-order spectra fits; Table S2: Bands parameters estimated from the Raman second-order spectra fits; Table S3: Peaks parameters and the atomic compositions estimated from the XPS spectra fits.
