# Supplementary material for: Spectral and Structural Properties of High-Quality Reduced Graphene Oxide Produced via a Simple Approach Using Tetraethylenepentamine
Source: Nanomaterials (Basel). 2022 Apr 7;12(8):1240. doi: 10.3390/nano12081240 (PMC9031607; doi:10.3390/nano12081240)
Supplement: Supplementary file 1 [file nanomaterials-12-01240-s001.zip › nanomaterials-1637572-supplementary.pdf]

Supplementary information

Spectral and Structural Properties of High-Quality Reduced Graphene Oxide Produced via a Simple Approach Using Tetraethylenepentamine

Table S1: Bands parameters estimated from the Raman first-order spectra fits

Sample	Band	Raman shift (cm^{-1})	Peak area (arb. units)	Peak%	I_D/I_G
**Graphite**	D	1350	1366	33.0	0.49
	D''				
	G	1581	2768	67.0	
	D'				
**GO**	D	1347	249105	63.7	2.19
	D''	1497	11949	3.1	
	G	1582	113728	29.1	
	D'	1615	16440	4.1	
**rGO-TEPA1**	D	1348	16899	60.9	2.16
	D''	1520	2238	8.1	
	G	1586	7837	28.2	
	D'	1627	780	2.8	
**rGO-TEPA2**	D	1344	14825	59.5	2.09
	D''	1528	2041	8.2	
	G	1586	7104	28.5	
	D'	1613	961	3.8	

Table S2: Bands parameters estimated from the Raman second-order spectra fits

Sample	Band	Raman shift (cm^{-1})	Peak area (arb. units)	Peak%	$I_{2D}/I_{D+D'}$
**Graphite**	2D	2700	4066	100.0	
	D+D'				
	2D'				
**GO**	2D	2700	94272	46.0	1.21
	D+D'	2934	78151	38.2	
	2D'	3166	32400	15.8	
**rGO-TEPA1**	2D	2693	6026	51.3	1.9
	D+D'	2909	3179	27.1	
	2D'	3140	2541	21.6	
**rGO-TEPA2**	2D	2701	9827	60.9	2.68
	D+D'	2922	3668	22.7	
	2D'	3153	2644	16.4	

Table S3: Peaks parameters and the atomic compositions estimated from the XPS spectra fits.

Sample	Peak	functional group	Binding energy (eV)	Peak area (arb. units)	at. %
GO	C 1s	C-C	284.8	6897	14.94
	C 1s	C-O	286.6	9026	19.55
	C 1s	C=O	288.4	5358	11.61
	C 1s	C(O)OH	290.5	1787	3.87
	O 1s	O-C=O	528.5	3680	7.97
	O 1s	C=O	529.8	6634	14.37
	O 1s	C-O	531.1	10926	23.67
	O 1s	C-O-C	533.4	1858	4.02
rGO-TEPA1	C 1s	C-NH_2	285.8	8831	17.54
	C 1s	C-C	284.7	8966	17.80
	C 1s	C-O	287.1	9592	19.05
	C 1s	C=O	289	4814	9.56
	C 1s	C(O)OH	291.9	2229	4.43
	O 1s	O-C=O	530.4	203	0.40
	O 1s	C=O	531.5	2744	5.45
	O 1s	C-O	532.8	5521	10.96
	O 1s	C-O-C	534.3	3430	6.81
	N 1s	N-H	399.3	312	0.62
	N 1s	N-H_2	400.3	1452	2.88
	N 1s	O=C-N	401.7	2267	4.50
rGO-TEPA2	C 1s	C-NH_2	286	7138	16.87
	C 1s	C-C	284.9	4911	11.61
	C 1s	C-O	287.1	7082	16.74
	C 1s	C=O	289	3225	7.62
	C 1s	C(O)OH	291.6	375	0.89
	O 1s	O-C=O	530.3	1079	2.55
	O 1s	C=O	531.6	5058	11.96
	O 1s	C-O	532.8	5274	12.47
	O 1s	C-O-C	534.4	2753	6.51
	N 1s	N-H	399.3	300	0.71
	N 1s	N-H_2	400	1408	3.33
	N 1s	O=C-N	401.2	3701	8.75
